# The second study of experimental human folate deficiency

**DOI:** 10.1186/2193-1801-3-719

**Published:** 2014-12-10

**Authors:** Joseph E Baggott, Tsunenobu Tamura

**Affiliations:** Department of Nutrition Sciences, University of Alabama at Birmingham, Birmingham, AL 35294 USA

## Dear Editor

We believed for over a half century that the landmark study by Herbert (1962) of experimental human folate deficiency is the one and only of its kind. However, there is a recent study by Golding (2014) published in *SpringerPlus* (2014, 3:442) reporting follow-up of haematological and biochemical changes secondary to a long-term intake of a low-folate diet. In both audacious studies, each author courageously subjected himself as an experimental subject and consumed foods thrice-boiled in a large quantity of water. The principal difference between the two studies was that it took only 18 weeks for Herbert (1962) to observed megaloblastic changes in the bone marrow, whereas it was not until 82 weeks for Golding (2014) to detect this most advanced haematological sign of folate deficiency.

Herein, to explain this startling difference, we propose two possible mechanisms. First, the difference may be explained by markedly higher baseline serum and erythrocyte folate concentrations (about 7 – 8 times higher than the cutoff for deficiency) in the study by Golding (2014) than those close to the cutoff points reported by Herbert (1962). These values are presented in Table 1, assuming that values by one of the assays in the recent study are believable. These suggest that the baseline total body folate stores must have been markedly different. If we simply calculate based on the duration until the end point of observing megaloblastic changes, the difference is about 4.6 fold (82/18). A vegetarian diet was consumed before the initiation of the experiment, which undoubtedly lead to above normal folate stores (Golding^2014^). Such high body stores may be common in the countries where the fortification of staple foods with folic acid is practiced, and Lin et al. (2004) reported that the total body folate store of adults in the US is 225 μmol which is considerably higher than previously believed.

**Table 1:** **Baseline and endpoint data of the subjects fed a low-folate diet**

Investigator	Herbert (1962)		Golding (2014)	
	Baseline	Endpoint^a^	Baseline	Endpoint
Weeks to observe megaloblastic changes^a^	18		82	
Body weight (kg) (kg body weight loss/week)	77	65 (0.67)	67	44 (0.28)
Erythrocyte (x10^8^/mm^3^)	5.75	4.5	5.2	2.1
Haemoglobin (g/100 mL)	15.5	13.9	16	8.9
Haematocrit (%)	48	42	45	24
Mean corpuscular volume (μm^3^)	84	92	84	116
Serum folate (ng/mL)	8.5^b^	< 1.0^b^	> 24^c^	< 1.0^c^
Erythrocyte folate (ng/mL)	150^b^	10^b^	1,318^c^	338^c^
Urinary formiminoglutamic acid (μg/12 hours)^d^	31	> 70	ND^e^	ND
Serum homocysteine (μmol/L)^d^	ND	ND	9.9	49.3
Vitamin C supplementation (mg/day)	70		500	
Vitamin B-12 supplementation (μg/day)	6		1000	

^a^Time point when megaloblastic changes were observed in bone marrow.

^b^Measured by *L. rhamnosus* microbiological assay.

^c^Measutred by protein-binding assay. We took values from only one assay method (Laboratory B).

^d^These test results are affected by both folate and vitamin B-12 status.

^e^Not determined.

Second, we postulate that the larger amount of ascorbate consumed in the recent study lead to a longer duration required to develop severe folate deficiency. Golding (2014) supplemented his experimental diet with a dose of 500 mg/day of ascorbate. On the other hand, Herbert (1962) took only 70 mg/day of ascorbate. It is important to note that in human vitamin C deficiency, megaloblastic changes have been observed, and these were responsive to the vitamin C supplementation (reviewed by Stokes et al.^1975^). In addition, Stokes et al. (1975) reported that a vitamin-C-deficient patient complicated with folate deficiency excreted only 10-formyl-folic acid (10-formyl-FA) in urine instead of fully reduced folates, such as 5-methyltetrahydrofolate (THF), 5-formyl-THF and 10-formyl-THF (Stokes et al.^1975^). 10-Formyl-FA (an oxidation product of 10-formyl-THF), is enzymatically inert in humans; it cannot be utilized for synthesis of purines and thymidylate, which are essential for maintaining normal haematological function. In this patient, urinary excretion of only 10-formyl-FA was not altered after an oral dose of 5-formyl-THF. However, after 150 mg/day of ascorbate treatment, 90% of urinary folate was 5-methyl-THF following another oral dose of 5-formyl-THF. These findings indicated that ascorbate protected labile fully-reduced folates from oxidation (Stokes et al.^1975^). Brown (1951) also reported that a folate-deficient patient responded haematologically to the administration of ascorbate alone. According to Levine et al. (1996), tissue ascorbate concentrations are saturated at an intake of 400 mg/day or more in humans. Although it is unknown whether the ascorbate saturation is essential to protect fully reduced folates from oxidation, a reduced rate of oxidation of 10-formyl-THF to 10-formyl-FA by excess ascorbate may preserve small amounts of 10-formyl-THF derived from small amounts of folate in the experimental diet.

We do not know how the difference between the large vitamin B-12 supplementation in the recent study protocol and that by Herbert (1962) may have affected clinical folate deficiency (Table 1). However, increased serum homocysteine is consistent with folate deficiency (Table 1).

We believe that both experimental findings by Golding (2014) and Herbert (1962) are valid. Difference between these studies might have resulted from a combination of the above mechanisms. Therefore, the data presented by Golding (2014) must be cautiously interpreted. The title of the study by Golding (2014) should have contained words clearly indicating that the subject had a high initial folate status and was saturated with ascorbate. A 500 mg/day intake of ascorbate and 1000 μg/day of vitamin B-12 can only be achieved by supplementation. The study design by Golding (2014) should have included a balanced supplementation of micronutrients at the level of generally recommended dietary intakes. We learned from these studies how difficult it is to perform reasonable experiments even after designing an extremely meticulous experimental protocol.
